# Substantial Achilles adaptation following strength training has no impact on tendon function during walking

**DOI:** 10.1371/journal.pone.0255221

**Published:** 2021-07-29

**Authors:** C. M. Waugh, A. Scott

**Affiliations:** Department of Physical Therapy, Faculty of Medicine, University of British Columbia, Vancouver, BC, Canada; National Tsing Hua University, TAIWAN

## Abstract

Tendons are responsive to mechanical loading and their properties are often the target of intervention programs. The tendon’s mechanical properties, particularly stiffness, also govern its function, therefore changes to these properties could have substantial influence on energy-saving mechanisms during activities utilizing the stretch-shortening cycle. We investigated Achilles tendon (AT) function *in vivo* during walking with respect to a training intervention that elicited significant increases in AT stiffness. 14 men and women completed 12-weeks of isometric plantarflexor strength training that increased AT stiffness, measured during isometric MVC, by ~31%. Before and after the intervention, participants walked shod at their preferred velocity on a fully-instrumented treadmill. Movement kinematics, kinetics and displacement of the gastrocnemius medialis muscle-tendon junction were captured synchronously using 3D motion capture and ultrasound imaging, respectively. A MANOVA test was used to examine changes in AT force, stress, strain, stiffness, Young’s modulus, hysteresis and strain energy, measured during walking, before and following strength training. All were non-significant for a main effect of time, therefore no follow-up statistical tests were conducted. Changes in joint kinematics, tendon strain, velocity, work and power and muscle activity during the stance phase were assessed with 1D statistical parametric mapping, all of which also demonstrated a lack of change in response to the intervention. This *in vivo* examination of tendon function in walking provides an important foundation for investigating the functional consequences of training adaptations. We found substantial increases in AT stiffness did not impact on tendon function during walking. AT stiffness measured during walking, however, was unchanged with training, which suggests that increases in stiffness may not be evident across the whole force-elongation relation, a finding which may help explain previously mixed intervention results and guide future investigations in the functional implications of tendon adaptation.

## Introduction

Bipedal locomotion is one of the most fundamental characteristics of humans [1] and one which has a large influence on quality of life, cognitive and physical health, social connections and independence as well as providing environmental benefits. The world over, people rely on walking to accomplish a wide range of tasks related to daily living, work and leisure activities, thus we seek to walk as economically as possible [2] by minimizing metabolic energy expenditure for a given movement speed or external power output [3]. Several energy-saving mechanisms have been identified to aid efficient locomotion, including the reutilisation of stored strain energy by tissues demonstrating elastic properties. Energy storing tendons, such as the Achilles, can act like springs by storing elastic energy when deformed by loads and returning that energy to the system during recoil. This process reduces the overall amount of energy required to perform mechanical work by reducing muscle metabolic work, which then lowers the metabolic cost of movement. It has been estimated that 6% of the total energy requirement of walking and 35% of that of running can be attributed to elastic energy storage and recoil of the Achilles tendon alone [4, 5].

To store meaningful amounts of elastic energy, tendons need to exhibit a certain amount of compliance—according to Hooke’s Law, a compliant tendon is able to store more elastic energy for a given force than a stiff tendon. Moreover, under optimum activation conditions, compliant tendons can facilitate greater muscle mechanical efficiency than stiff tendons [6]. However, compliant tendons also have lower restoring forces, which might attenuate propulsion, and are exposed to greater strains which increases injury risk [7, 8]. A tendon stiffness which accommodates these conflicting requirements is necessary for economical movement. As tendon stiffness and muscle strength are positively correlated [9], concurrent increases in both may maintain strain levels [10], but a net increase in elastic strain energy storage might be observed given the greater forces involved in stretching a stiffer tendon. Whilst the return of strain energy with tendon recoil is thought to contribute to reducing the energy cost of locomotion, not all of the energy that is stored in the elastic structures is returned; tendons also exhibit viscoelastic properties, and some of the energy initially stored is lost as heat (i.e. hysteresis). *In vivo* estimations of Achilles tendon hysteresis range between 2%–49% in young adults, depending on the methodology [4, 11, 12] and can decrease by as much as 35% with plyometric training [13]. Improving the efficiency of the storage-recoil process through training would be of huge functional importance and likely contribute to greater movement economy.

Muscular efficiency is also a major determinant of movement economy and is influenced by the attached tendon’s compliance [6, 14]. Tendon compliance can influence muscle efficiency by uncoupling muscle fascicle shortening from the shortening pattern of the muscle-tendon unit and performing the majority of a movement’s required length change [15]. This feature enables cross-bridge cycling [and therefore heat energy liberated in relation to shortening; 16] and fascicle shortening velocity to be minimized and enhance force production [17]—all whilst providing an opportunity to keep the muscle at an optimal length for force production [18]. These energy-saving mechanisms are maximised when movement is performed at preferred frequencies [19, 20].

It is well known that tendon tissue is responsive to mechanical loading and unloading. Due to the influence of the tendon on muscle mechanics and timing of activation [21], power output [6], joint stiffness and reflex response [22] movement frequency [20] and force production [23, 24], its mechanical properties are often the target of intervention programs, particularly those with an emphasis on rehabilitation, injury prevention and optimisation of sporting performance. However, the tendon’s mechanical properties also govern its function, thus tendon adaptation as a result of training or pathological conditions may influence energy-saving mechanisms during activities utilizing the stretch-shortening cycle [21, 25, 26].

Differences in tendon behaviour during walking have been noted with pathology and chronic adaptation. For example, diabetics demonstrate greater Achilles tendon (AT) stiffness, which attenuated AT length changes during walking [27, 28]. Long-term high heel users demonstrate increased tendon stiffness and reduced tendon strain [29] which shifts the stretch distribution of the muscle-tendon unit during walking towards larger muscle fascicle strains as the tendon resists lengthening [30]. Irrespective of the aetiology, changes in tendon properties may alter movement efficiency and injury risk, hence we sought to better understand the impact of changes in tendon properties on tendon function during the important and relevant task of walking. We investigated AT function *in vivo* before and after a 12-week strength training intervention that elicited a mean tendon stiffness increase of ~31% using the same relative force levels pre-post training [31]. We hypothesized that energy-saving characteristics (e.g. hysteresis, elastic strain energy reutilization) would be enhanced with such stiffness increases during walking, and suspected that such improvements would alter dynamic tendon strain, velocity and power. To our knowledge, this study is the first to examine *in vivo* tendon function in walking in relation to a training intervention and so provides an important foundation for investigating the functional consequences of training adaptations.

## Materials & methods

This study was granted institutional ethical approval by The University of British Columbia’s Clinical Research Ethics Board (CREB), approval number H15-00043. All study testing conformed to the guidelines set out in the Declaration of Helsinki.

### Study participants and recruitment

The present data were captured from participants whose tendon adaptation in response to the training interventions outlined below have been published previously [31]. Eighteen individuals volunteered to participate in the current study, of which 14 adult men (n = 7, age 30.1±7.9 y, weight 83.2±6.0 kg, height 181.9±5.8 cm) and women (n = 7, age 29.9±5.2 y, weight 61.3±7.2 kg, height 164.5±7.5 cm) completed the training. All participants were free from known neuromuscular and musculoskeletal disorders, were physically active (undertook moderate to vigorous physical activity at least 3 times per week, confirmed with the International Physical Activity Questionnaire; IPAQ), had not participated in a structured resistance training program targeting the lower leg in the last 12 months and had not previously experienced an AT injury. Participants provided fully informed written consent for participation in the study and were made aware of their right to withdraw from the study at any time, without penalty. All study testing took place at laboratories within the Centre for Hip Health and Mobility, Vancouver BC, Canada.

### Training intervention

An overview of the plantarflexor training intervention are presented here in brief; additional details can be found in Waugh et al. [31]. Plantar flexion training was performed on an isokinetic dynamometer (Biodex 3, Medical Systems, New York, USA). The lateral malleolus of the fibula was aligned with the rotational axis of the dynamometer arm and the relative internal hip, knee and ankle angles were approximately 95°, 180° and 0°, respectively. Training sessions were performed three times per week for a 12-week period [32]. Each session consisted of 5 sets of 10 isometric contractions at a load equal to 90% MVC [33]. Each session was completed in the presence of a research team member to a pre-programmed protocol providing visual and audio cues for maintaining consistent strain frequency and rate across sessions. Consecutive sets were separated by a 90-s rest period. Plantarflexor MVC was re-tested at the beginning of each training week and training loads modified accordingly. Different training protocols were assigned to leg side using a within-subject randomization stratified according to leg dominance, for the hypothesis of examining the influence of rest duration on tendon adaptation [31]. The long rest training (LRT) protocol differed from the short rest training (SRT) protocol only in the duration of rest time between consecutive contractions; LRT incorporated a 10-s rest between each contraction whereas the SRT was a 3-s rest. Training volume was identical between protocols. AT stiffness pre- and post-intervention was calculated from AT deformation over 25–90% of pre-training isometric MVC values; these MVCs were performed over a 3-s ramp. The training intervention induced a mean AT stiffness increase of ~31%; no statistical differences were found in the magnitude of AT stiffness adaptation or of any other biomechanical property examined between training protocols [31].

### Pre- and post-training data acquisition

Participants visited the laboratory prior to commencing the 12-week training intervention, during which they were familiarized with the study protocol, data collection techniques and equipment. Participants were asked to refrain from heavy exercise for 48 hours prior to testing sessions and ingesting anti-inflammatory drugs and changing physical activity habits throughout the training period. In addition to research variables, participant mass and height were acquired before and after the intervention.

### Experimental protocol

Every study participant had prior experience of treadmill locomotion, although few had experience with a split-belt treadmill. To familiarise participants with the experimental set-up, participants walked shod in their chosen athletic footwear on a fully-instrumented treadmill (Bertec, OH, USA) at their preferred treadmill-adjusted walking velocity for a 10-minute period [34]. Preferred walking velocity was obtained by incrementally increasing belt speed from a slow to fast walk, and asking for feedback on comfort. Once a fast speed had been determined, the belt speed was incrementally lowered until a comfortable speed was reached (preferred walking velocity was described to participants as the speed they might walk to work, to a public transit stop, etc). Mean walking velocity was 1.1 ± 0.2 m/s. Participants were asked to focus on a vertical line positioned approximately 3 meters in front of the treadmill which was aligned with the split in the treadmill belt. This helped participants to maintain the correct position on the treadmill whilst diverting attention away from performing the walking task (i.e. aiding walking autonomy). The same footwear and walking velocities were used in pre- and post-training assessments.

### Gait kinematics and kinetics

**Fig 1:**
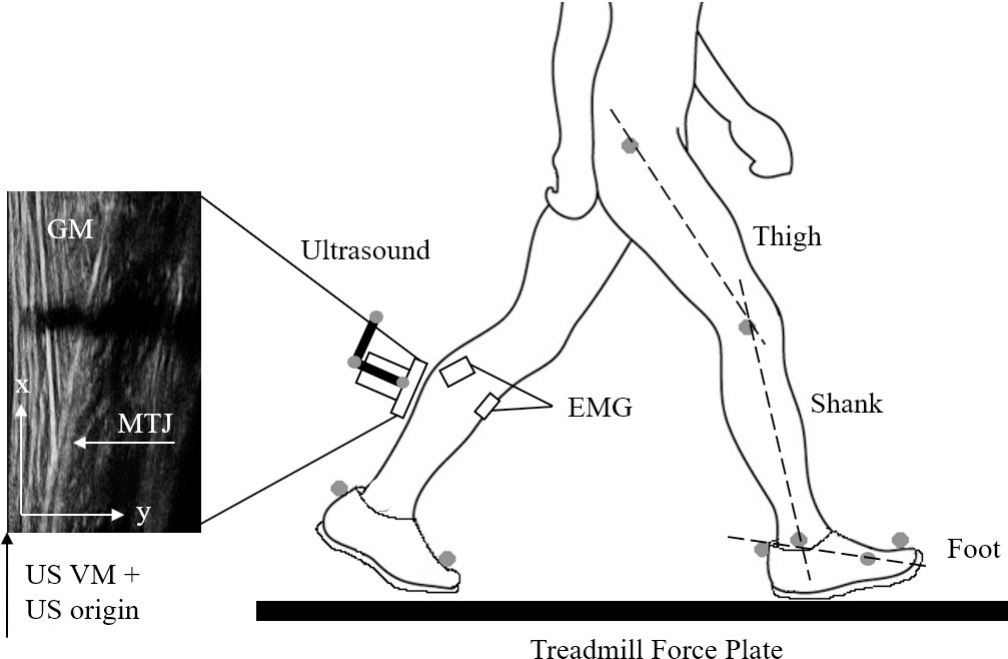
Experimental set-up and illustration of ultrasound (US) configuration for calculating GM MTJ coordinates. Grey dots on lower body represent placement of motion capture markers; ultrasound graphic includes 3-marker rigid body from which a virtual marker (VM) was constructed at the edge of scanning interface. The VM corresponded with the origin of the US reference frame and was used to transform the 2D coordinates of the MTJ to the motion capture’s 3D coordinate system, from which tendon length was estimated.

Reflective markers were placed on the greater trochanter, lateral femoral condyles, medial and lateral malleolus, proximal calcaneus, first and fifth metatarsal heads, toe and ultrasound probe handle (Fig 1). As footwear obscured the proximal calcaneus (reference point for AT insertion, determined from palpation of the area), the calcaneus marker was positioned on the shoe at the same height as the proximal calcaneus during quiet standing. The distance from the skin to the marker centroid was measured with callipers and accounted for by amending its coordinates with reference to the ankle joint centre of rotation location, which was defined as the midpoint between the medial and lateral malleoli. Markers were tracked in a pre-calibrated space using a passive 3D motion capture system (Cortex v3.6, Motion Analysis, Santa Rosa, CA) to provide movement kinematics. Marker coordinates were sampled at 150 Hz and were smoothed using a zero-lag fourth order Butterworth filter at 7 Hz. Knee angle was calculated as the acute angle between the greater trochanter-lateral femoral condyle-lateral malleolus markers, whilst ankle angle was defined as the acute angle between the lateral femoral condyle-lateral malleolus-fifth metatarsal head markers. The treadmill was fully integrated within the motion capture system and ground reaction forces were synchronously sampled at 1500 Hz alongside other analog signals (EMG, synchronizing trigger) using a 16-bit A/D card (NI USB-6218, National Instruments, Austin TX, USA).

### Electromyographic measurement of muscle activity

Myoelectric activity was measured from the *gastrocnemius medialis* (GM) and *tibialis anterior* (TA) muscle bellies. The electrode site was shaved and the skin lightly abraded prior to cleansing with an alcohol-based antiseptic wipe. Bipolar electrodes (Bagnioli-8, Delsys Inc., Natick MA, USA) were positioned approximately in parallel with the orientation of the underlying fascicles. A reference electrode was placed over the right patella to account for background noise. EMG signals were digitally filtered using a 20–500 Hz 4th order Butterworth band pass filter prior to calculating the root-mean-square (RMS) envelope using a 34ms (51 samples) moving window length. RMS EMG was normalized to peak RMS EMG amplitude during isometric MVC trials [31, 35]. Electrodes were not removed between performing MVC and walking activities.

### Ultrasound

As the training protocol was designed to target the gastrocnemii (see ‘*Training Intervention’* for details*)* we visualized displacement of the GM muscle-tendon junction (MTJ) with B-mode ultrasound (RP Sonix, Ultrasonix, Burnaby BC, Canada; 60 mm linear array probe, 57 Hz sampling frequency, 12 Mhz scanning frequency). The probe was positioned just medial to the septum with the gastrocnemius lateralis, and orientated to clearly visualize both the superficial and deep GM aponeurosis (Fig 1). The probe was fixed in position using a custom-made probe holder and elastic taping. Ultrasound was independently acquired and synchronized with a trigger pulse sent to all acquisition devices. GM MTJ displacement was manually identified in each frame using open-source software (Tracker v4.91, opensourcephysics.com). Data were low-pass filtered using a fourth-order, zero-lag Butterworth filter with a 3.5-Hz cut-off frequency, determined by residual analysis.

To obtain tendon length, assigning the GM MTJ 3D coordinates was required. Using the 3D marker cluster fixed to the US probe as a reference, a virtual marker was created in the motion capture software at one edge of the ultrasound probe’s scanning interface (together, the virtual marker and 3D marker cluster were considered a single rigid body). Given its location, the virtual marker could also be identified in the US image (Fig 1) and was used to transform the 2D coordinates of the GM MTJ to the motion capture’s 3D coordinate system. Instantaneous AT length was defined here as the linear distance between the GM MTJ and calcaneus coordinates.

### Calculation of variables used for assessing tendon function

All variables were derived using a custom written analysis program (Matlab v.2014a, Mathworks, Cambridge, UK). Gait cycle events were determined from the ground reaction force (GRF) vertical component. Initial (heel) contact and toe-off were defined as the points at which the baseline signal during the swing phase 1) exceeded and 2) returned to within 2 standard deviations above the baseline, respectively. Eight successive stance phases free from GRF contamination by the contralateral leg were identified and used for data analysis. Ankle joint plantarflexor moment about the ankle centre of rotation was estimated using an inverse dynamics approach. AT force was calculated as the ratio of instantaneous plantarflexor moment to instantaneous AT moment arm length. AT moment arm length was estimated as the perpendicular distance from the ankle centre of rotation to the tendon line of action (i.e. tendon length vector, see following paragraph). To calculate tendon stress, peak AT force was divided by subject-specific AT cross sectional area [CSA; 31]. Peak strain was calculated as the largest change in length from AT length at heel strike during the stance phase only. Stiffness and Young’s modulus were also calculated from 50–100% of the AT forces experienced during walking, to identify whether the large increases in AT stiffness with training were evident during walking.

Tendon length change is presented in reference to its length at initial contact. Tendon velocity was calculated as the first derivative of displacement with respect to ground contact time. Tendon work was estimated as the first derivative of velocity with respect to displacement. Tendon power was calculated by multiplying AT force by velocity at each time point of the stance phase. To assess hysteresis, a work loop was constructed from the tendon force-elongation relationship; force was normalised to body weight (N). AT hysteresis was calculated as the difference between the area under the loading and unloading portions of the tendon work loop, expressed as a percentage of the area under the loading curve only. Strain energy returned (*Utot*, *J*) was calculated using *Utot* = 0.5 (*s*^2^/*E*) *Vt* × *c*, where *s* is peak tendon stress during walking (MPa), *E* is Young’s modulus, *Vt* is tendon volume (m^3^) and c is a constant reflecting the ratio of stored energy returned to the system after hysteresis (i.e. c = 0.4 for 60% hysteresis). *E* and *Vt* were obtained from previously acquired data [31].

### Data analysis

Scalar data were analysed using SPSS statistical software (IBM SPSS v25.0, IBM Corp., Armonk, NY, USA). Pre-training differences in peak AT force, stress, strain, stiffness, Young’s modulus, hysteresis and tendon strain energy storage between legs assigned to the LRT or SRT interventions were assessed using an initial MANOVA. A two-way repeated measures MANOVA was then used to assess the effects of time (pre; post) and training type (SRT; LRT) on these dependent variables. In the case of significance, individual univariate tests were completed and followed by Bonferroni post hoc t-tests. Pre-post changes in stride frequency and stride length were examined with paired t-tests to identify whether they may have influenced the functional outcome measures. Significance was accepted at p≤0.05.

Time-series data were carried out using the open-source spm1d code (v.M.0.4.6, www.spm1d.org) in Matlab (R2014a, The Mathworks Inc, Natick, MA). Pre-training differences in tendon displacement, velocity, work and power curves between training groups were examined with independent t-tests using statistical parametric mapping (SPM). Pre-post changes to these variables as well as AT force and RMS EMG were also assessed with paired t-tests using SPM (all tests with *α*-level of 0.05). Pre-post changes in ankle and knee kinematics were also examined with SPM to identify whether they may have influenced functional outcome measures.

## Results

Of the participants who completed the study, 6 participants (3 men, 3 women) performed LRT on their right leg and 8 participants (4 men, 4 women) on their left leg. The contralateral limb received the SRT protocol. All participants self-reported to be right-leg dominant. Training compliance was 95%; no participant missed more than 4 training sessions throughout the training period.

There were no pre-training differences found between training groups for any dependent variable measured, as assessed by the initial MANOVA (Wilks’ Lambda = 0.783; F = 0.792, df_7,20,_ *p* = 0.603). The repeated measures MANOVA revealed no main effect of training group (Wilks’ Lambda = 0.792; F = 0.751, df_7,20,_ *p* = 0.633) or time (Wilks’ Lambda = 0.820; F = 0.628, df_7,20_, *p* = 0.727), and no significant group × time interaction (Wilks’ Lambda = 0.551; F = 2.330, df_7,20_, *p* = 0.065) on pre-post scalar dependant variables, therefore data were combined henceforth. Stride frequency and length were unchanged (p = 0.64–0.76). Pre-post data, % pre-post change and effect sizes are presented in Table 1. All outcome variables had effect sizes ≤0.5, indicating that our strength training intervention had a minimal effect on the variables we examined [36].

**Table 1:** Temporal, dimensional and functional dependant variables calculated pre- and post-training intervention.

Variable		Pre	Post	% Δ	Cohen’s *d*
Stride Frequency (Hz)	Mean	1.12	1.13	0.3	0.04
	s.d.	0.08	0.06		
Stride length (m)	Mean	1.00	1.02	1.9	0.17
	s.d.	0.23	0.19		
AT length,	Mean	206.1	205.1	-0.5	0.06
resting (mm)	s.d.	33.0	39.7		
AT length,	Mean	191.3	196.2	3.5	0.46
heel-strike (mm)	s.d.	38.8	34.0		
AT CSA (mm^2^)	Mean	60.3	60.8	0.8	0.09
	s.d.	13.7	14.9		
Peak AT Force (N)	Mean	1965.7	2012.7	2.4	0.16
	s.d.	483.9	514.5		
Strain (%)	Mean	6.6	6.3	-4.4	0.06
	s.d.	3.3	2.9		
Stress (MPa)	Mean	33.2	33.5	1.13	0.06
	s.d.	7.7	6.9		
Stiffness (N/mm)	Mean	101.8	107.3	5.4	0.15
	s.d.	37.8	42.3		
Young’s modulus	Mean	351.7	365.5	3.9	0.07
	s.d.	141.8	148.5		
Hysteresis (%)	Mean	40.7	41.0	0.6	0.02
	s.d.	9.7	11.8		
Strain Energy (J)	Mean	12.2	12.5	2.4	0.40
	s.d.	5.6	6.7		

Data are averaged from the two training modalities after no significant differences were found between. Cohen’s *d* effect sizes calculated from paired pre-post data differences [36].

**Fig 2:**
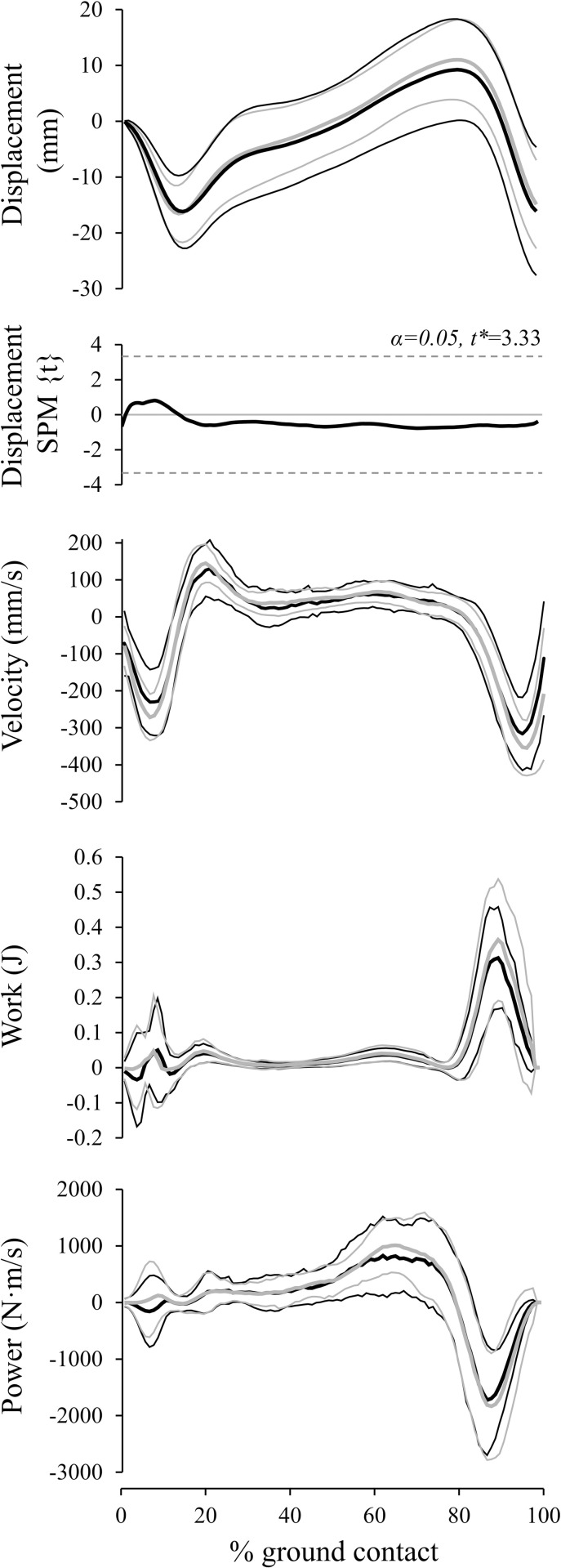
Tendon displacement, velocity, work, and power before (black, thick) and after (grey, thick) training protocols (±1 s.d. thin lines). Dependent variables represent averaged data from all participants during the stance phase of the gait cycle. SPM analysis demonstrates lack of change in displacement from pre-post training.

**Fig 3:**
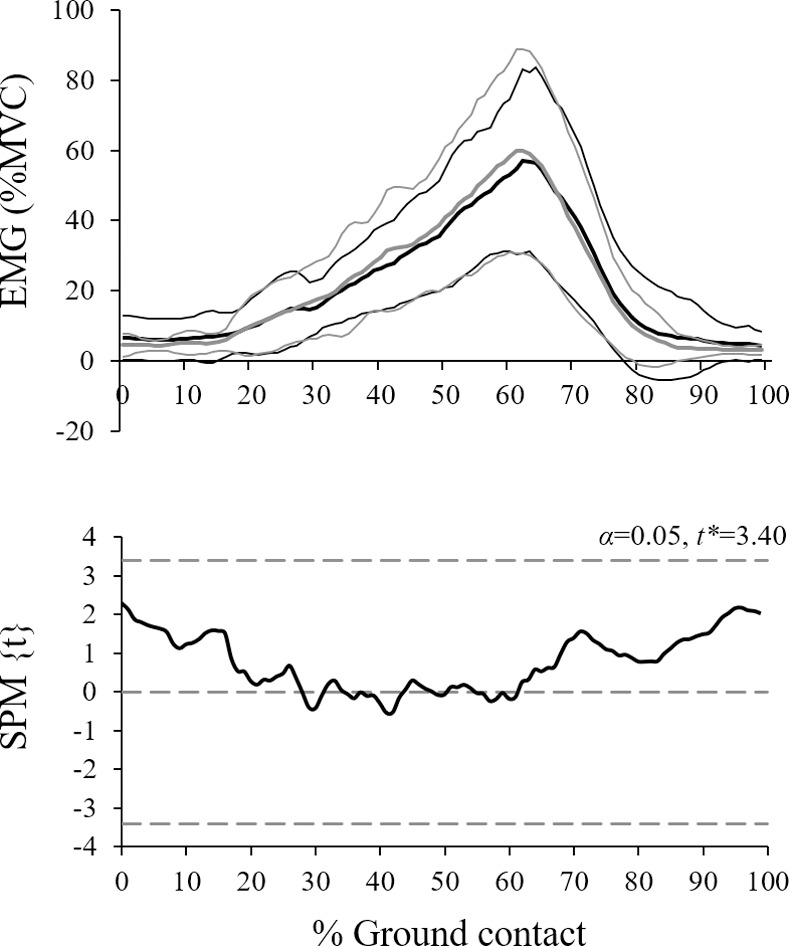
Mean ± s.d. (thick and thin lines, respectively) GM EMG amplitude pre (black) and post (grey) across the ground contact phase. EMG amplitude is relative to the maximum amplitude achieved across all plantarflexion MVC trials. SPM analysis demonstrates no change in muscle activation during walking as a result of the training interventions.

Results from the SPM analyses demonstrated that the tendon displacement, velocity, work and power curves were highly similar between groups pre-training and did not change significantly as a result of either plantarflexor training intervention (Fig 2). Differences in the heel-strike (absolute) length of the tendon were unchanged pre-post training, therefore there were no pre-post differences present at heel-strike which may have obscured these results. Similarly, tendon force, joint kinematics and RMS EMG amplitude were not notably altered from either intervention. GM muscle activation over the ground contact period and associated SPM analysis are illustrated in Fig 3.

**Fig 4:**
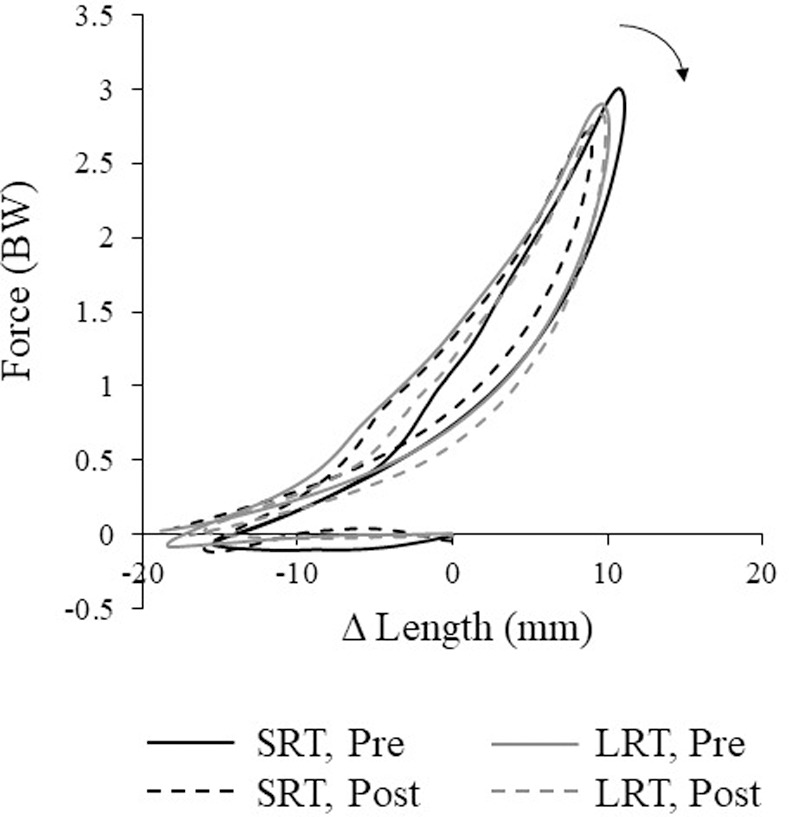
Tendon work-loop, normalized to unit length at heel strike and body weight (BW) before (solid) and after (dashed) training protocols. Arrow depicts loop direction.

Tendon displacement replicated previously published reports [e.g. 19, 27, 37, 38]. Peak shortening velocities and power outputs for all components were achieved during the push off phase (Fig 2). As AT force and length increased synchronously and peak AT strain occurred approximately with peak force, the clockwise tendon work-loop created indicated net work absorption (Fig 4).

## Discussion

Tendon stiffness is a functionally important property whose widespread function has implications for both elite performance as well as injury prevention and falls. As such, it is often the focus of studies relating to human movement. From the abundance of research available on tendon adaptation with training, it is generally accepted that a training program involving mechanical loading will alter the biomechanical properties of the targeted tendon. However, there is little research on how this impacts *in vivo* tendon function, despite reports of tendon pathology and long-term adaptation affecting tendon biomechanical properties and function in walking. As strength training is so broadly recommended, we examined tendon function in walking for its applicability to the general population. Whilst not exhaustive, a host of variables were examined in an attempt to identify the impact of a ~31% increase in AT stiffness on tendon function during preferred-velocity walking. Interestingly, very little appeared to have been impacted as a result of this change, implying that tendon function during walking is relatively insensitive to substantial increases in tendon stiffness measured at high force levels. Importantly, such increases in tendon stiffness did not seem to impair tendon function.

The AT stiffness calculated from AT forces during walking was unaltered after training, despite large increases in AT stiffness observed with isometric contractions. A change in stiffness would have required a reduction in elongation or increase in AT force during walking, but neither were observed to change in walking; this is contrast to our findings during MVC, where displacement decreased and AT force increased significantly [31]. Despite large strength gains from the training protocol, AT forces during walking did not change–likely a result of maintained subject weight and pre-post walking velocity [39]. By controlling walking velocity, stride and joint kinematics and AT loading rates appeared constrained, all of which could influence AT stiffness.

Our findings indicate that increases in AT stiffness may not be evident across the whole force-elongation relation. Indeed, use of a force range common to both tasks may have allowed for a better initial comparison. Peak forces during walking are suggested to be 36–52% of those during MVC [40, 41], so the force range used in estimating tendon stiffness during walking would perhaps correlate to ~20–50% of MVC forces. Our walking force-elongation relation showed significant linearity (R^2^ = 0.95 ± 0.05) but may have included slightly more curvilinear data than with 25–90% MVC [R2 = 0.96 ± 0.04; 31]. i.e. stiffness calculated with walking may involve more of the toe-region of the force-elongation relation than with MVC. This is supported by *in vitro* studies which suggest the toe-region (associated with collagen uncrimping) may account for tendon strains <4% [42, 43] and *in vivo* studies that demonstrate maximum AT strain in walking is ~4% [41, 44].

Tendon adaptation to high loads and strains is complex and may involve structural, mechanical and/or chemical changes to the tendon hierarchy. Changes that have been linked to improved mechanical properties include increased fibril size, changes in the non-collagenous matrix, increased collagen cross-linking and better fibre organization. Importantly, such modifications will affect tendon extension mechanics, which differ with strain level. At low forces, the toe-region is primarily associated with collagen un-crimping and alignment, whist at higher forces the linear region involves inter-fascicle sliding and physical deformation of the collagen structures [e.g. 45, 46]. It is possible that these mechanisms are affected unequally with training, or that only certain mechanisms affect tendon stiffness during particular loading conditions [e.g. 47]. For example, linear region stiffness is determined by intermolecular connectivity that is directly dependent on cross-links type and density [48]. There is evidence that cross-links type and density is altered/increased with training, affecting the fascicle sliding and physical extension mechanisms activate at higher forces (MVC). While crimp morphology has been shown to adapt to the altered functional and mechanical demands of exercise [49–51], the relevance of these changes with respect to tendon mechanical properties are largely unknown. Based on this evidence, it is feasible that the primary extension mechanisms allowing a greater tendon strain in MVC may be different to walking and respond differently to training. Such mechanisms may help to explain why differences in AT stiffness were found with MVC but not walking.

Tendon dimensions were unaffected by the training intervention [31], therefore peak stress and Young’s modulus were also unchanged from pre-post training. Given the lack of change in variables used to calculate strain energy, it is not surprising that the amount of elastic energy stored and returned in any given cycle did not change either. This finding is in keeping with modelling data which suggests that increases in tendon stiffness of the magnitude seen in our study would not affect tendon energy storage appreciably during walking, but may be more impactful during higher impact activities [21, 52]. Decreases in tendon hysteresis have been targeted by interventions previously, as this would be beneficial for efficient movement by reducing the amount of energy lost as heat, but have thus far proved inconclusive. Stretching interventions demonstrate promising reductions in hysteresis [53, 54], while resistance [53, 55] and plyometric [13, 56] interventions report mixed results.

The results from dynamic *in vivo* studies do not calculate hysteresis or strain energy *per se*, but tendon strain patterns during functional tasks are examined, and so elastic energy storage and return can be discussed. Findings of lower tendon recoil [26], higher tendon shortening velocity [57] and no change in tendon strain patterns [25, 58]—as found presently—with concurrent increases in AT stiffness (measured with MVC) demonstrate a lack of consensus on how increased tendon stiffness may impact on energy saving mechanisms. Albracht and Arampatzis [25] found significant improvements in running economy after a 14-week training intervention which resulted in ~16% increase in AT stiffness measured with MVC. They did not find alterations in AT strain patterns after the intervention, thus to achieve the same AT elongation during running and account for the reduction in energy cost of the movement, estimated that AT force should be about 25% higher post-intervention for the same running speed. However, Werkhausen et al., [26] found AT force did not increase during running in spite of an 18% increase in AT stiffness and unaltered AT strain patterns after training despite a reduction in AT strain observed during isometric MVC. Running kinematics were unchanged in both studies. The authors suggest that training adaptations alter muscle-tendon interaction in more complex ways than suggested by in-series models, for example with changes in biaxial strain patterns of the muscle-tendon complex or in neuromuscular control. It is certainly plausible that changes in muscle architecture and mechanics that occur with training interventions modulate uniaxial tendon loading and therefore stiffness [59] and should be investigated implicitly in future. We found no appreciable change in agonist muscle activity in our study, however Hirayama et al., [57], found increased agonist (and decreased antagonist) activity during the breaking phase of depth jumps after a plyometric training intervention which increased AT stiffness (measured with MVC) by 35%. Whilst movement kinematics were not controlled, these findings suggest that changes in tendon mechanical properties may be associated with the optimization of neuromuscular control of stretch-shortening activities.

The tendon is considered to act like a spring in bouncing gaits and impart significant energy-saving mechanisms, yet oftentimes it is deemed that energy storage and return is limited during walking. Alexander (1991) suggested that reutilization of elastic strain energy would not likely occur during walking due to its rigid inverted pendulum design. The emergence of a bipedal spring-mass model [60], which incorporates spring-like elements into the inverted pendulum design by allowing the stance leg to compress and recoil, challenges this view. Fukunaga et al. [37] reported tendon strain patterns in walking demonstrated spring-like storage and release of elastic strain energy, thus substantial reutilization of elastic strain energy should be accomplished after all. Indeed, we and others estimate significant energy recovery by the AT during walking [4, 12, 61]. Nonetheless, AT loading rates are lower during walking, which may exacerbate the tendon’s viscoelastic properties and contribute to greater hysteresis values in comparison with bouncing gaits [e.g. 11, 62]. Zelik and Franz [12] found similar hysteresis values to this study (37±27% for 1.0 m/s walking velocity) when using a similar methodology, and also report walking velocity-dependant hysteresis trends (49±17% at 0.75 m/s; 11.3±38.6% at 1.25 m/s). Pointing at the insensitivity of hysteresis to loading rates in isolated tendon, the authors instead hypothesise methodological factors were behind the larger hysteresis values. Certainly there are methodological considerations, particularly relevant to the calculation of hysteresis [63, 64], that would be at play in our study. Whilst we used instantaneous moment arm lengths, thus accounting for angle and load-dependence, to improve AT force estimates currently, tendon strain may be underestimated by modelling the AT as a linear structure. Likely the biggest limitation, however, would be in our US sampling rate, which may have exacerbated issues relating to force-elongation desynchronization. Whilst the same methods were used for pre- and post-training, desynchronization could occur in either direction and therefore we cannot say that any errors are systematic. As such, our hysteresis findings should be interpreted cautiously.

Tendon behaviour during functional activities has typically focussed on young, healthy individuals, but differences have been noted in populations demonstrating changes in the tendon matrix structure and composition as a result of pathological conditions or long-term adaptation. For example, diabetic patients demonstrate attenuated tendon length changes, greater stiffness and hysteresis during walking [27, 28, 65], which are likely an outcome of accelerated and excessive glycation of tendon tissue collagen [66]. In the normal body, advanced glycation end products (AGEs) accumulate slowly over time, thus they are also a likely contributor to the altered tendon behaviour observed in older adults compared to younger counterparts [38]. Degenerative structural changes associated with tendinopathy reduces the biomechanical integrity of the tendon and results in reduced tendon stiffness [e.g. 67], which also affects tendon function. Arya and Kulig [67] found individuals with Achilles tendinopathy demonstrated 15% higher strains, whilst Child et al. [68] found AT strain was significantly higher in athletes with Achilles tendinopathy (5.2±2.6%) compared to control athletes (3.4±1.8%). In addition to clinical populations, chronic involvement in activities may impart a lasting effect on muscle-tendon mechanics. Long-term high heel use is associated with increased tendon stiffness [29] and therefore larger muscle fascicle strains [30] as the tendon resists lengthening during walking activities. Whether pathological or not, changes in tendon properties may compromise muscle efficiency [21] whilst increasing the risk of strain-related injuries, hence future research should focus on the functional outcomes of tendon adaptation.

## Conclusion

Whilst there now seems to be more focus on the functional implications of tendon adaptation, the relationship between tendon properties and improvements in movement economy remain unclear; it seems that there are still many outstanding questions and methodological considerations that must be overcome. To our knowledge, this study is the first to examine *in vivo* tendon function during walking in relation to a training intervention designed to elicit significant tendon adaptation. It acknowledges that substantial increases in AT stiffness as a result of a training intervention, measured via MVC, may not be detectable in activities which involve different portions of the force-elongation relation. Our findings provide the foundation for examining the functional implications of tendon adaptation during higher impact activities in future research. Despite measuring a 31% increase in AT stiffness with MVC as a result of a 12-week strength training intervention, we did not identify any significant changes in tendon mechanical properties or function during walking. Importantly, substantial increases in tendon stiffness as a result of such interventions did not have a negative impact on tendon function during walking activities. This finding may be particularly relevant for clinical and ageing populations, where walking capacity is a critical contributor to quality of life, yet tendon properties can be optimised to enhance rapid force production (i.e. RFD, EMD) required for falls prevention and improved balance and stability.

## Supporting information

S1 File (XLSX)
